# A non-linear optimisation method to extract summary statistics from Kaplan-Meier survival plots using the published *P* value

**DOI:** 10.1186/s12874-020-01092-x

**Published:** 2020-10-30

**Authors:** Andrew F. Irvine, Sara Waise, Edward W. Green, Beth Stuart

**Affiliations:** 1 Faculty of Medicine, University of Southampton, Southampton, UK; 2 Present Address: Department of Pathology and Data Analytics, University of Leeds, Leeds, UK; 3 The German Cancer Research Centre (DKFZ), Heidelberg, Germany

**Keywords:** Survival analysis, Kaplan-Meier plot, Meta-analysis, Algorithm, Life table, Non-linear optimisation

## Abstract

**Background:**

Meta-analyses of studies evaluating survival (time-to-event) outcomes are a powerful technique to assess the strength of evidence for a given disease or treatment. However, these studies rely on the adequate reporting of summary statistics in the source articles to facilitate further analysis. Unfortunately, many studies, especially within the field of prognostic research do not report such statistics, making secondary analyses challenging. Consequently, methods have been developed to infer missing statistics from the commonly published Kaplan-Meier (KM) plots but are liable to error especially when the published number at risk is not included.

**Methods:**

We therefore developed a method using non-linear optimisation (nlopt) that only requires the KM plot and the commonly published *P* value to better estimate the underlying censoring pattern. We use this information to then calculate the natural logarithm of the hazard ratio (ln (HR)) and its variance (var) ln (HR), statistics important for meta-analyses.

**Results:**

We compared this method to the Parmar method which also does not require the number at risk to be published. In a validation set consisting of 13 KM studies, a statistically significant improvement in calculating ln (HR) when using an exact *P* value was obtained (mean absolute error 0.014 vs 0.077, *P* = 0.003). Thus, when the true HR has a value of 1.5, inference of the HR using the proposed method would set limits between 1.49/1.52, an improvement of the 1.39/1.62 limits obtained using the Parmar method. We also used Monte Carlo simulations to establish recommendations for the number and positioning of points required for the method.

**Conclusion:**

The proposed non-linear optimisation method is an improvement on the existing method when only a KM plot and *P* value are included and as such will enhance the accuracy of meta-analyses performed for studies analysing time-to-event outcomes. The nlopt source code is available, as is a simple-to-use web implementation of the method.

## Background

In many medical studies, the main outcome measured is the time until a specific event occurs, otherwise known as survival or time-to-event data. Analysing this data requires specific statistical methods to account for the fact that only some individuals will experience the event, a process known as censoring [1]. Censoring might occur because an individual has not experienced the event being measured by the end of the study, they are lost to follow-up during the study, or they experience another event which makes further follow-up impossible. Common methods used to analyse survival data which adjust for censoring include Kaplan-Meier plots, log-rank tests and Cox (proportional hazards) regression [1].

With many studies reporting survival data published each year, systematic reviews and meta-analyses have become increasingly commonplace, assessing the strength of evidence accrued in aggregate across multiple studies analysing the same factor (e.g. therapeutic intervention or the prognostic role of a particular biomarker). The advantages of meta-analyses include increasing power, improving precision and providing an opportunity to deal with conflicting claims [2].

Although the gold standard for meta-analysis is using individual patient data (IPD), allowing a much more flexible approach to analysing survival data [3], the IPD is not always available and attempts to acquire it can involve a significant investment in time and cost [4]. As such, a meta-analysis based on aggregate data is a reasonable alternative that often generates similar conclusions when compared with an IPD meta-analysis, especially when comparing summary statistics such as ln (HR) and the variance (var.) of ln (HR) [5]. However, aggregate data meta-analyses (hereafter referred to as simply meta-analysis) can be challenging when primary studies fail to report sufficient data and statistics, leading to the exclusion of such studies from secondary analyses. This is of particular concern in research examining the clinical significance of prognostic factors, in which independent studies often report inconsistent or conflicting findings [2], and therefore where secondary analyses would be the most valuable. As a consequence, meta-analyses are often unable to conclude with confidence the role of a particular prognostic factor [6].

Work to improve the quality of prognostic studies has established a simplified checklist of recommendations derived from the REMARK criteria for reporting time-to-event data [7]. Items 15 and 17 of the REMARK criteria highlight the importance of publishing the univariate hazard ratio (HR), confidence intervals (CI) and statistical significance (*P* value) for time-to-event data. In the case where prognostic studies or any other study-type publish the above, ln (HR) and var. ln (HR) can then be used to calculate an average of ln (HR) with the weights inversely proportional to var. ln (HR) [8].

Although these studies might also report other summary statistics associated with survival, including single points estimates such as the median survival, these have been shown not to be a reliable marker of time-to-event outcomes [9] and thus the recommendation is still to use ln (HR) and var. ln (HR) where available.

Despite the above criteria, not all studies report these statistics [10], especially those published before the establishment of the REMARK criteria in 2005 [11]. As a result, several methods have been developed to try and accommodate data from primary studies which did not originally report either ln (HR) or var. ln (HR) (more commonly both) into meta-analyses. They are all based on estimating these statistics from more commonly reported information included in primary studies involving time-to-event data such as the number of events in each arm, number at risk values, and the KM survival curve itself. In particular, the methods of Parmar et al. [8] have become a widely utilised method of inferring ln (HR) and var. ln (HR) from primary literature. In this study, a hierarchy of methods depending on the reported evidence was presented, including both simple calculations to obtain the var. ln (HR) from reported confidence intervals and more resource-intensive methods to calculate ln (HR) and var. ln (HR) from Kaplan-Meier (KM) plots. For the latter, survival probabilities from each arm of a KM plot are first extracted at specific time points. Estimations of the minimum and maximum follow-up times as well as an assumption that patients are censored at a constant rate throughout the study period are then used to calculate ln (HR) and var. ln (HR) by creating pooled estimates.

Since this study was published, other methods have been developed that further increase the accuracy of estimating summary statistics by incorporating additional information, in particular the number at risk data that should be included underneath Kaplan-Meier plots. For instance, Vale et al. [12] used these values to better estimate the censoring pattern in calculating the odds ratios at fixed time points. In an attempt to establish a framework of common time intervals across trials, Williamson et al. [13] developed a method using the number at risk to improve estimation of ln (HR) by assuming that censoring was constant within time intervals rather than across the whole study (as in the Parmar method). However, by aiming to establish common intervals between trials, some of the survival probabilities were not included in the analysis, therefore Hoyle and Henley [14] extended Williamson’s method to use all the survival probabilities within the time interval stipulated by the number at risk to improve the estimation of ln (HR) and var. ln (HR) [15].

The most recent advance published by Guyot et al. [16] uses the number at risk and total number of events and an iterative numerical approach to more accurately identify the underlying censoring pattern and thus a more accurate estimation of the IPD and summary statistics [15]. However, when these two pieces of information are not included, the method, as acknowledged by the authors performs poorly [16].

Indeed, all these methods, with the exception of the Parmar method, rely on the number at risk data being published to give accurate estimates of ln (HR) and var. ln (HR). Whilst the inclusion of this information is more commonplace than it used to be [12], it can vary significantly depending on the field. Oncology randomised-controlled trials tend to be better at reporting associated information, including the number at risk, compared to biomarker studies [10, 15] although this is certainly not the case in every published study [15]. We therefore set out to develop a novel method that did not require inclusion of the number at risk, instead utilising the commonly published *P*-value to improve the accuracy of estimating censoring patterns in primary datasets.

Here we report the implementation of such a method using non-linear optimisation to estimate ln (HR) and var. ln (HR) and show it improves on estimations using the Parmar method. We believe this method will be useful for meta-analysis studies, in particular for incorporating studies where the number at risk is not published. We have made our method available as an R script, and a simple-to-use graphical web-app for researchers to use.

## Methods

### Derivation of the underlying equations and description of the algorithm

In collecting survival data, study participants either experience the measured event or are censored at a specific time point. The Kaplan-Meier survival estimate (eq. 1) used to create a Kaplan-Meier plot is simply the probability of surviving from one interval to the next multiplied together to give the cumulative survival probability [1].

- The Kaplan-Meier survival estimate

1 $$ S\left({t}_j\right)=S\left({t}_{j-1}\right)\left(1-\frac{e_j}{n_j}\right) $$

Where *S*(*t*_*j*_) is the probability of being alive at time *t*_*j*_, *e*_*j*_ is the number of events at *t*_*j*_ and *n*_*j*_ is the number of patients alive just before *t*_*j*_.

If a study is carried out and all participants experience the event such that no censoring occurs, this survival estimate is simply the ratio of the number of individuals event free at time *t* divided by the number of people who entered the study. Thus, in cases where there is censoring, a combination of the survival probability and an accurate estimation of the number of censored participants would enable the complete Kaplan-Meier survival table to be reconstructed. In the Kaplan-Meier survival estimate, the number of censored participants at *t*_*j*_ is not formally defined but is contained within the number at risk and can be separated out by defining *n*_*j*_ (eq. 2).

- Define *n*_*j*_

2 $$ {n}_j={n}_{j-1}-\left({censor}_{j-1}+{e}_{j-1}\right) $$

With the censor value isolated, the Kaplan-Meier survival estimate can now be re-arranged to calculate the number of events at *t*_*j*_ based on the survival probability extracted from the KM plot.

- Rearrange (1) to solve for *e*_*j*_

3 $$ {e}_j={n}_j-\left\{{n}_j\left[\frac{S\left({t}_j\right)}{S\left({t}_{j-1}\right)}\right]\right\} $$

Finally, substitute (eq. 2) into (eq. 3) to derive an equation that calculates the number of events based on the survival probability and the level of censoring at *t*_*j*_.

- Substitute n_j_ with (2) in (3) to re-define *e*_*j*_

4 $$ {e}_j=\left[{n}_{j-1}-\left({censor}_{j-1}+{e}_{j-1}\right)\right]-\left\{\left[{n}_{j-1}-\left({censor}_{j-1}+{e}_{j-1}\right)\right]\left(\frac{S\left({t}_j\right)}{S\left({t}_{j-1}\right)}\right)\right\} $$

With the above equation, it is now possible to create a table detailing the number of events, degree of censoring, number at risk as well as the expected number of events for each time point. This allows calculation of the HR using (eq. 5) as well as calculating the degree of significance with the log-rank test, a widely used method of generating a *P* value that is commonly published alongside a KM plot.

The hazard ratio function

5 $$ HR=\frac{O_1/{E}_1}{O_2/{E}_2} $$

Where *O*_*1/2*_ and *E*_*1/2*_ are the observed and expected total number of events for group 1 and 2, respectively.

The next step in the analysis relies on the fact the log-rank statistic commonly used to calculate the level of significance in a KM plot is approximately distributed as a chi-square test statistic which itself contains information directly related to the observed and expected number of events in each arm (eq. 6).

The chi-squared test statistic

6 $$ {X}^2=\sum \limits_{i=1}^g\frac{{\left({O}_i-{E}_i\right)}^2}{E_i} $$

Where *O*_*i*_ and *E*_*i*_ are the observed and expected total number of events for group *i*, respectively, with g the number of groups.

A work-through of the above is presented in Additional file 1 to illustrate the method.

At this point, the censoring pattern is still unknown but there is a fixed point i.e. the Chi-squared test statistic which is directly related to these values through the full survival table calculated for the log-rank test. Each censoring value thus becomes an unknown number to be solved to satisfy the chi-square test statistic to calculate an optimal solution. Such problems can be solved using non-linear optimisation (nlopt). Nlopt addresses general non-linear optimisation problems of the form:

minimise *f*(*x*) x in R^n^

So that

$$ g(x)\le 0,\kern0.50em h(x)=0,\kern0.50em lb\le x\le ub $$

where f is the objective function to be minimised and x represents the n optimisation parameters. Lb and ub represent lower and upper limits for x. *g*(*x*) represent the inequality constraint(s), and *h*(*x*) represents the equality constraint(s).

In the case of using nlopt to solve this problem, our objective value to solve is the chi-squared test statistic calculated as in (eq. 6). Where x is all the censor values from each time point to be solved. We specify lower and upper bounds for x as 0 and infinity respectively. Thus, our objective function, *f*(*x*) is all the steps in the table above which contribute to determine this.

The primary equality constraint, *h*(*x*) is:

7 $$ \sum \limits_{i=1}^g\frac{{\left( Oi- Ei\right)}^2}{Ei}-\mathrm{known}\ \mathrm{chi}\ \mathrm{squared}\ \mathrm{test}\ \mathrm{statistic}=0 $$

In the case of a non-exact *P* value, our equality constraint becomes an inequality constraint when the P value is expressed as < than a certain value

8 $$ \sum \limits_{i=1}^g\frac{{\left( Oi- Ei\right)}^2}{Ei}-\mathrm{known}\ \mathrm{chi}\ \mathrm{squared}\ \mathrm{test}\ \mathrm{statistic}\le 0 $$

In the case where the *P* value is expressed as more than 0.05, or non-significant, we reverse the sign for the chi-square statistic to set a minimum value for a right-tailed P value as *p* = 0.05 (*X*^2^ =3.841) whilst we also set a maximum limit of *p* = 0.95 (*X*^2^ =3.9e-3).

$$ \sum \limits_{i=1}^g\frac{{\left( Oi- Ei\right)}^2}{Ei}+\mathrm{known}\ \mathrm{chi}\ \mathrm{squared}\ \mathrm{test}\ \mathrm{statistic}\le 0 $$

Our two additional equality constraints for which *h*(*x*) = 0 are the total number of deaths and censoring values for arm 1 and arm 2 and should equal the starting number at risk for each group and represent two additional equality constraints and are represented below for arm 1 and arm 2:

9 $$ \sum \limits_{j=1}^nC1j+E1j-\mathrm{N}{1}_0 $$

10 $$ \sum \limits_{j=1}^nC2j+E2j-\mathrm{N}{2}_0 $$

Where, C1 and C2 are censor values at time j in arm 1 and 2 respectively; E1 and E2 are events at time j in arm 1 and 2 respectively and N1_0_ and N2_0_ the starting number at risk in arm 1 and 2. Where n is the number of time points.

Many software packages exist to solve this problem, but the R interface to NLopt (an open-source library for non-linear optimisation algorithms [17]) is simple to use and can be written using R (Version 3.5.2, Vienna, Austria). There are several optimisation routines available within the NLopt wrapper, but *slsqp*, a sequential (least squares) quadratic programming (SQP) algorithm was chosen as it supports both equality and inequality constraints. The R scripts can be found at the following online repository: https://gitlab.com/EdGreen21/irvinekm

### Model assumptions

Since the tests (log-rank and Cox proportional hazards) used to generate the *P* values required for this method are based on proportional hazards (PH) [18], it should be assumed that this method will perform most optimally under the assumption of proportional hazards. However, unless the assumption of PH is strongly violated, we believe the nlopt method will still perform well. See Discussion for more detail.

### Extraction of X,Y coordinates from published KM plots

Points were extracted using the Fiji distribution of ImageJ (version 1.52p; NIH, USA). The KM plot is first loaded into ImageJ and a rectangle corresponding to a known X,Y area drawn within the figure to calibrate the axes using the Figure Calibration Plugin (Frederic V. Hessman, University of Gottingen). To aid reading points from the plot, vertical lines corresponding to specified times were automatically drawn on the figure using a custom script written in Fiji. Due to the design of the method, every time value should have a corresponding y value from arm 1 and 2. The corresponding data should be stored in three columns: Time (t), Arm 1 (y_1_) Arm 2 (y_2_). By definition, t_0_ should be 0 and the corresponding survival probability, 1 for each arm. Time values should not be duplicated i.e. they must increase in number each iteration although y1/y2 values can stay the same but obviously not increase (decreasing monotonicity function). Given this, a function to check the user input follows these rules has been included in the R script and online version of the method to ensure spurious results are not outputted by the nlopt method in case a user inadvertently inputs data which invalidates the rules above. This is similar to the input checks included with the writing of the Guyot method as a Stata function [19].

### Extracting ln(HR) and var. ln(HR) from the Parmar paper

Figures 2 and 3 from the paper published by Parmar [4] are X, Y graphs plotting ln (HR) and var. ln (HR) values obtained from a comparison of the survival curve method and a direct or indirect estimation of these values. The X axis plots ((survival curve + direct/indirect)/2) whilst the Y axis plots (survival curve -direct/indirect). The same method described for extracting values from KM plots was used to extract the X, Y co-ordinates for these figures. To extract these values from the above, the equations were re-arranged to: survival curve ln(HR) or var ln(HR) = $x+\frac{y}{2}$; Direct/indirect ln(HR) or var = $x-\frac{y}{2}$

Where *x* and *y* represent the extracted values from Figs. 2 and 3 from Parmar et al. [8].

The mean absolute error (MAE) were then calculated as below.

### Calculation of mean absolute error and analysis of statistical significance

The MAE for ln (HR) and var. ln (HR) were calculated by subtracting the calculated values from the known, published values and the absolute value taken. The mean percentage absolute values were similarly calculated. A one-way ANOVA with Kruskal-Wallis multiple comparisons test was used to assess the statistical significance in Prism (GraphPad, Version 8).

### Evaluating the number and position of points required for an optimal solution

To assess the number of points required for an optimal solution using nlopt, the individual patient data (IPD) from three studies was used [20–22]. The Kaplan-Meier survival probability table was first constructed using the “*Survival*” package in R and the actual time and survival probabilities every time an event or censoring occurred (i.e. the KM survival table) for each arm stored as vectors. A random time value was then taken from one of the survival curves and the corresponding survival value at that specific time value selected. If no identical value was available from the other curve (which was occasionally the case as different groups often experience events at slightly different times), the maximum value either side of this X value was chosen. This in effect mirrors the process required to read the survival probability off a KM plot at different time points. This was iterated at 5-point intervals and 100 Monte Carlo simulations carried out using the nlopt method to determine ln (HR) and var. ln (HR) as described. In total, 50 points were used for two studies [20, 22] and 30 for the remaining study which represented the maximum number of timepoints in the actual dataset [21]. Summary statistics from these results were then calculated and the means and standard deviations plotted using Prism (GraphPad, Version 8).

The weighted simulation experiments were carried out using a total of 30 points distributed according to specific weights within the KM plot split into three equal sectors by time according to Table 3.

### Benchmarking of the nlopt method

**Fig. 1:**
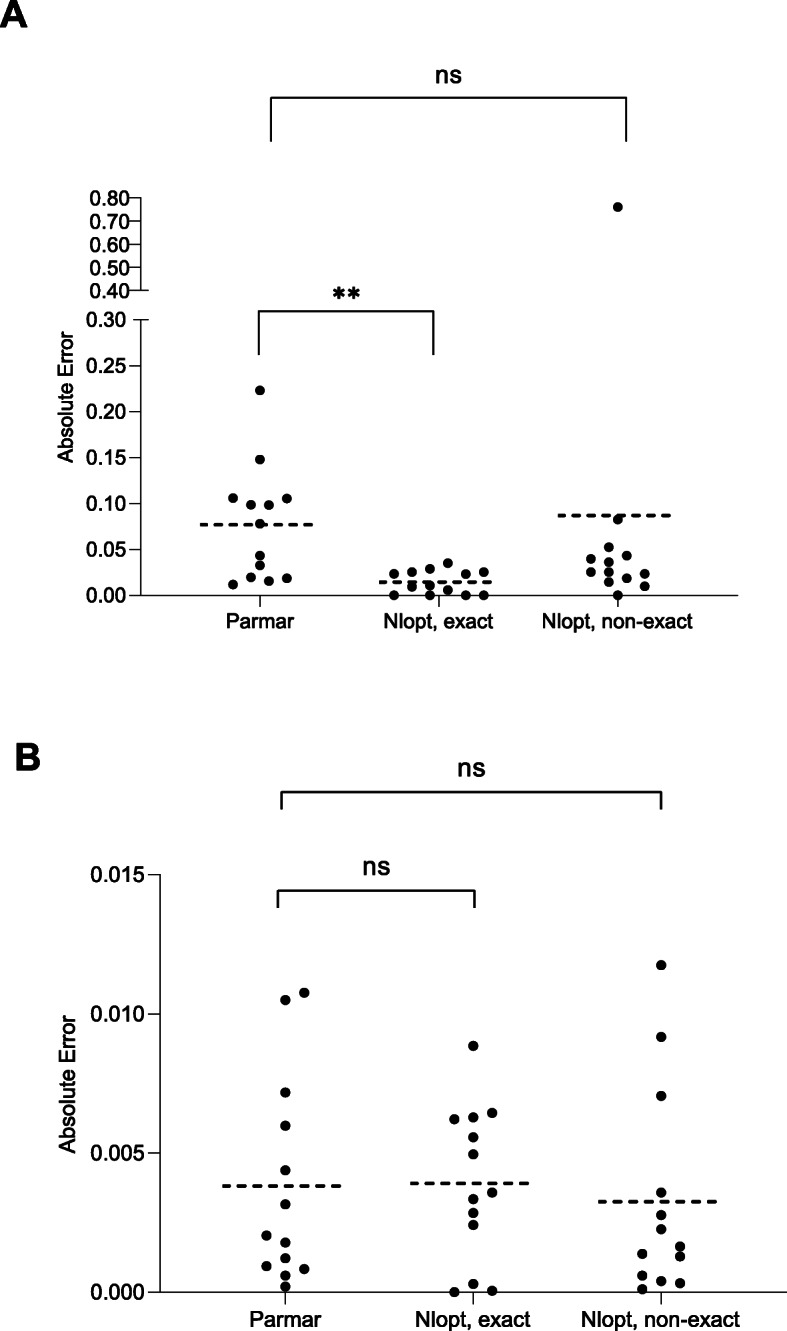
A comparison of the absolute error associated with ln (HR) and var. ln (HR) calculated using the Parmar and nlopt method (exact and non-exact *P* value). The ln (HR) and var. ln (HR) were estimated from 13 KM plots using the Parmar and nlopt method (exact and non-exact *P* value). The absolute error was then calculated from the known, actual values published in each article. The graph represents ln (HR) (**a**) and var. ln (HR) (**b**) of each individual study calculated using each method with the horizontal line in each column equal to the mean. The statistical significance was assessed using a one-way ANOVA with Kruskal-Wallis multiple comparisons test. ** = *P* < 0.01, ns = *P* > 0.05

The benchmarking of the nlopt method using an exact and non-exact *P* value was carried out on a laptop computer with an Intel® Core™ i5–6200 CPU @ 2.30Ghz with 8GB of installed RAM. Benchmarking was calculated as the time difference between “Sys.time()” in R at the start and end of carrying out 100 iterations of each of the 13 KM plots described to validate the nlopt method in Fig. 1. The average of these times was taken and plotted for each KM plot.

## Results

### Rationale for developing a new method

As is widely acknowledged, many primary studies presenting time-to-event data do not report the necessary statistics to allow their inclusion in an aggregate meta-analysis [2]. However, while reviewing the literature on prognostic factors in lung cancer for a meta-analysis [23] we found numerous primary studies publish KM plots and a *P* value of unadjusted estimates. To further assess how common various scenarios of data reporting are, we carried out a secondary analysis of a published meta-analysis on prognostic factors in oesophageal adenocarcinoma [24]. This published meta-analysis excluded studies where ln (HR) and var. ln (HR) were not explicitly stated but made the entire database of screened studies available online. Of the 36 studies which were excluded due to a lack of these summary statistics, we encountered five different scenarios of reported data (Table 1).

**Table 1:** Summary of reported data scenarios from McCormick Matthews [24]

Scenario	Reported Data	Number	Required Method
1	i) *P* value/chi-square statistic for ln (HR) or HR ii) Total number of events	6 (17%)	Equations 8 and 10, Parmar [8]
2	i) IPD	0 (0%)	Available statistical methods
3	i) KM survival plot ii) Number at risk included at regular intervals	4 (11%)	Estimation from the KM plots, Guyot [16]
4a	i) KM survival plot ii) No total number of events iii) No number at risk included iv) *P*-value/chi-square statistic for ln (HR) or HR (exact)	19 (53%)	Estimations from the KM plots, Parmar [8]
4b	v) *P*-value/chi-square statistic for ln (HR) or HR (non-exact)	4 (11%)	
5	i) KM survival plot ii) No total number of events iii) No number at risk included iv) No *p* value	3 (8%)	Estimations from the KM plots, Parmar [8]
	**Total**	**36 (100%)**	

The frequency of reported data scenarios from a meta-analysis published by McCormick Matthews et al. [24] in which studies did not explicitly state both the ln (HR) and var. ln (HR). The required equations to extract ln (HR) and var. ln (HR) given that set of data is also given

In total, 17% of studies reported a *P* value calculated using log-rank/Cox regression tests and the total number of events (scenario 1), whilst no study included the individual patient data (IPD). However, in the majority of cases (64%) a KM survival plot with associated *P* value but no number at risk or total number of events was included (scenario 4). This analysis therefore confirmed that a large number of studies fail to directly report the necessary statistics to carry out a subsequent meta-analysis whilst a common scenario of data reporting in these instances is a KM plot and associated *P* value but no other information.

A number of methods exist to extract summary statistics in such scenarios. The method developed by Guyot et al. [16] can be used when a KM plot is included but for a good degree of accuracy, as acknowledged by the authors, the number at risk and total number of events also need to be reported. In the above analysis, only 11% of studies included this information. Otherwise, the method by Parmar et al. [8] can be used when a KM plot is included with or without a *P* value since it is not a requirement of the analysis. However, as the *P* value/chi-squared statistic is inherently related to the hazard ratio, it was theorised that this could be included in any reverse engineering of the KM plot to more accurately estimate ln (HR) and var. ln (HR) by better predicting the underlying censoring pattern. We therefore established a method based on non-linear optimisation (nlopt) using the survival probabilities from the KM plot and associated *P* value.

### Validating the non-linear optimisation algorithm

To first validate the nlopt method, thirteen KM plots from 11 different studies were identified in a range of articles published between 1999 and 2019 with a median study length of 60 months (range 1–120 months) (Additional File 2). The time and survival probability for each KM plot were extracted using publicly available open-source software as described in the Methods section, and the nlopt method used to calculate the summary statistics for each KM plot. The resulting estimations of ln (HR) and var. ln (HR) were compared to those obtained using the method outlined by Parmar et al. [8] (Fig. 1a, b) which as previously stated, does not require the number at risk to be published. The only requirement for including a study in this set was that they also directly reported ln (HR) and var. ln (HR) so the method could be validated. Unfortunately, only a single study tested the assumption of PH (Additional File 2); an issue well acknowledged in the literature [25]. The full set of results is recorded in Additional File 3 with a summary of the mean absolute errors (MAE) and mean % absolute errors shown in Table 2.

**Table 2:** Comparison of the mean absolute error and mean % absolute error using the Parmar method and nlopt method (exact and non-exact *P* value) described in this study

Method	Mean Absolute Error (95% CIs)		Mean % Absolute Error (95% CIs)	
	Ln (HR)	Var ln (HR)	Ln (HR)	Var ln (HR)
1. Nlopt method: exact *P* value	0.014 (0.007–0.022)	0.0039 (0.0022–0.0056)	6.63% (2.69–10.57%)	20.12% (5.51–34.73%)
2. Nlopt method: non-exact *P* value	0.087 (−0.036–0.210)	0.0033 (0.0010–0.0055)	27.37% (8.69–46.05%)	17.30% (3.68–30.91%)
3. Parmar	0.077 (0.039–0.115)	0.0038 (0.0016–0.0060)	43.47% (11.18–75.77%)	20.06% (6.50–33.62%)

The nlopt method using a exact and non-exact *P* value were compared with the Parmar method using a validation dataset of 13 KM plots. The mean absolute error and mean % absolute error for ln (HR) and var. ln (HR) were calculated for each method

Two scenarios for reported data were analysed using the nlopt method, one where the *P* value is and is not exactly stated thus corresponding to scenario 4(a) and 4(b) in Table 1 respectively. In the case of an exact *P* value, the nlopt method yielded a significantly improved mean absolute error (MAE) of 0.014 for ln (HR) compared to a MAE of 0.077 using the Parmar method (*P* = 0.003; Fig. 1a) or a mean % absolute error of 6.63% vs 43.47%. The validation of the Parmar method in this study was similar to that reported in their original study which gave a MAE of 0.079 [8]. Thus, for a HR of 1.5, reconstructed HRs differing by a factor of 1.08 and 1.01 would be expected i.e. 1.39/1.62 or 1.49/1.52 for the Parmar and nlopt method, respectively.

As outlined in Table 1, studies occasionally report a *P* value as a non-exact value such as < 0.01 or > 0.05. Although this is less common (Table 1: 4/23, non-exact *P* value vs 19/23, exact *P* value) it is of interest to establish how the nlopt method would perform in these cases. Such a scenario significantly changes the nlopt method as the objective value to solve for becomes an inequality and not an equality constraint as used for an exact *P* value.

To model the scenario where a *P* value is not explicitly stated but expressed as less than a certain value, the same thirteen KM plots were analysed but instead of the exact *P* value used for the analysis, a value one “scale” higher was chosen (Additional file 3). For example, if the exact *P* value was 0.01 or 0.005, then a non-exact value of < 0.05 or < 0.01 was chosen as the minimum value for the non-linear optimisation. This approach was taken as it likely reflects the situation in published studies where “scales” of non-exact *P* values are often quoted i.e. < 0.001 or < 0.01 rather than the exact *P* values themselves. In cases where the *P* value was > 0.05, or otherwise ‘non-significant’, lower and upper limits of the *P* value, i.e. 0.05 < *p* ≤ 0.95 were used. Although the nlopt method using a non-exact *P* value generally performed well (Fig. 1a), a single outlier from Breslow et al. [26] significantly skewed the results which meant the MAE was similar to that of Parmar (MAE, 0.087 vs 0.077, *P* = 0.6735). In the case of this specific example, the actual *P* value was significantly lower than quoted on the KM plot (*P* < 0.001 vs *P* = 4.47E-39). As might be expected, estimations of ln (HR) improved dependent on their proximity to the true *P* value (Additional File 4).

The variance of ln (HR) was also calculated using each of the methods and compared to the actual published values (Additional file 3). Unlike the results for ln (HR), the MAEs were similar with values of 0.0038, 0.0039 and 0.0033 for the Parmar, exact and non-exact nlopt methods, respectively, which were not statistically significant (*P* > 0.05; Fig. 1b).

### Evaluating the number of points required for an optimal solution

We next analysed the number of points required to obtain an optimal solution for a particular KM plot. To answer this question, three studies analysing survival data which had also made the complete IPD accessible alongside the survival analysis were chosen [20–22]. Studies with IPD were used to ensure that there was no inherent error associated with reading the survival probabilities off the KM plot whilst having access to all the time points and the corresponding survival probabilities meant a truly random set of points could be used for the multiple simulations required to test the method.

To assess the number of points required, a set number of random time values ranging from 5 to 50 for two of the studies and 5 to 30 (this represented the entirety of all the timepoints) for the other study at 5-point intervals were uniformly sampled along the entire timeframe. The corresponding survival probabilities at each time point were then calculated for both arms of the KM plot and 100 Monte Carlo simulations carried out using the nlopt method to obtain the mean and standard deviation of ln (HR) and var. ln (HR).

**Fig. 2:**
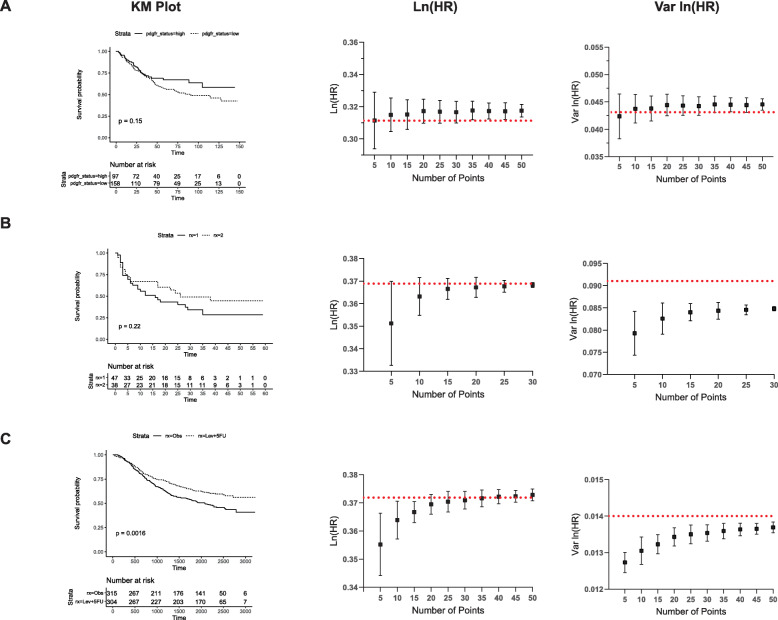
Determining the number of points to calculate an optimal solution using the nlopt method for an exact *P* value. The KM survival probabilities were extracted from three published studies. A set number of points, ranging from 5 to 50 (**a**, **c**) and 5–30 (**b**), were extracted from the KM survival probabilities and these points used to calculate ln (HR) (middle panel) and var. ln (HR) (right panel) using the nlopt method with an exact *P* value. One hundred Monte Carlo simulations were carried out and the mean and associated standard deviation plotted. The red, dashed horizontal line represents the actual value from the published study

We first examined a study in which the majority of events occurred at a largely constant rate during the first third of the time course (Fig. 2a) [20]. In such a scenario, the mean ln (HR) and var. ln (HR) approximated the true values with as few as 5 points although more points were required to minimise the standard deviation. Fig. 2b shows a KM plot in which the majority of events are clustered in the first half of the time series [21], and Fig. 2c a case where events are dispersed across the entire time series [22]. In these cases, estimating ln (HR) and var. ln (HR) from such curves requires more points to be extracted from the KM plot, with estimates for Fig. 2b stabilising around 15 points and Fig. 2c around 30, with additional points further reducing the variance of the estimates in these Monte Carlo simulations.

**Fig. 3:**
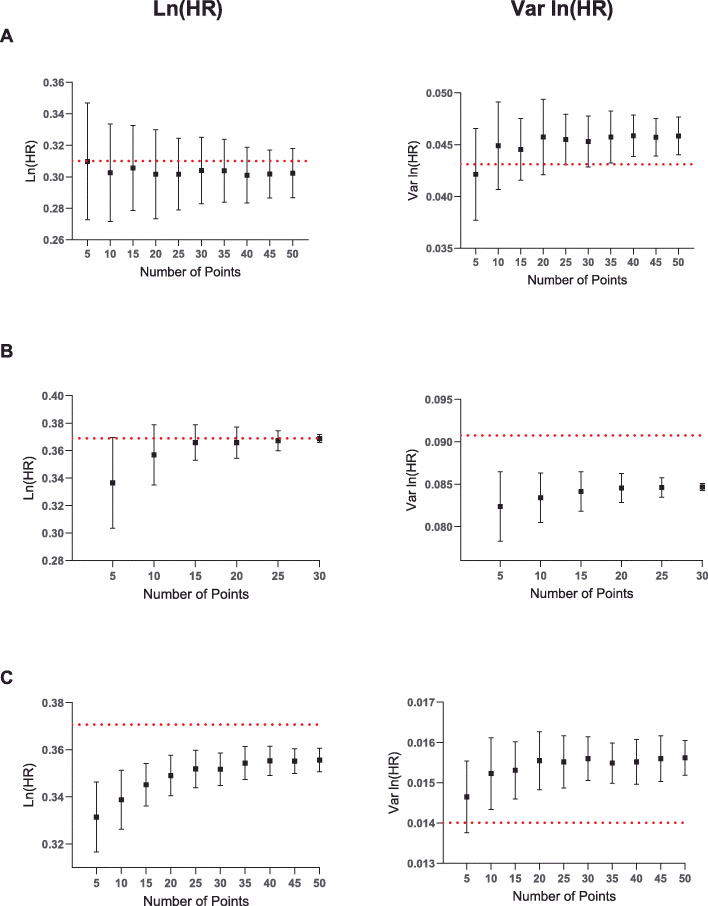
Determining the number of points to calculate an optimal solution using the nlopt method for a non-exact *P* value. The KM survival probabilities from the same studies as in Fig. 2 were used to calculate ln (HR) (left panel) and var. ln (HR) (right panel) using the nlopt method with a non-exact *P* value. One hundred Monte Carlo simulations were carried out and the mean and associated standard deviation plotted. The red, dashed horizontal line represents the actual value from the published study

The same analysis was also carried out with a non-exact *P* value to determine if more points would be required to achieve a better estimate of the summary statistics. Interestingly, the trend for each of three datasets analysed for ln (HR) and var. ln (HR) was in fact very similar (Fig. 3a-c). However, in the case of the last study (Fig. 3c), these estimates were less accurate whilst the standard deviations in the first and third studies were significantly larger than for an exact *P* value (Fig. 3a, c). Importantly, this analysis therefore shows that taking more points in the case of a non-exact *P* value does not improve the accuracy of the estimation.

### Assessing how the weighting of points in a KM plot affects the estimation of ln (HR) and var. ln (HR)

We next examined where these points should be distributed along the curve to ensure an optimal solution. To carry out this experiment, the timeframe of the first study (Fig. 2a) was split into three equal sectors. A total of 30 points were then distributed in a weighted manner within each of these sectors to create a set of weighted-time series according to Table 3 and 100 Monte Carlo simulations carried out.

**Table 3:** The weighting of points in each sector used to assess how the positioning of points affects estimates of ln (HR) and var. ln (HR)

Sets, Study 1	Sector 1 (0–50 months)	Sector 2 (51–100 months)	Sector 3 (101–150 months)	Total Number of Points
Uniform	10	10	10	30
Weight 1	20	5	5	30
Weight 2	5	20	5	30
Weight 3	5	5	20	30

The maximum timeframe was split into three equal sectors as described. A series of weighted sets were created for each study based on distributing 30 points at specific weights across each of the sectors

**Fig. 4:**
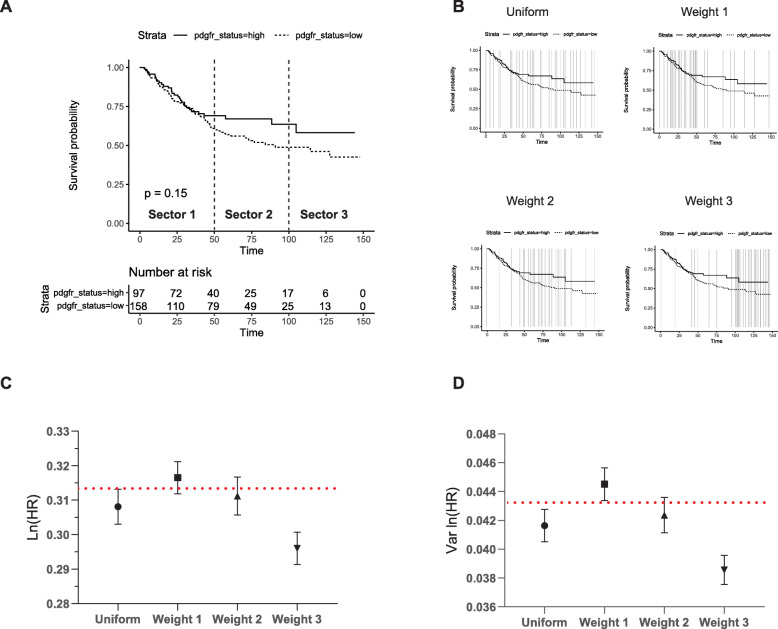
Assessing how the weighting of points in a KM plot affects the estimation of ln (HR) and var. ln (HR). The KM plot from Fig. 2a was used to determine the effect of weighting points at different areas of the KM plot. The KM plot was split into three equally divided sectors along the entire timeframe of study (**a**). A total of thirty points was randomly distributed according to the weights described in Table 3. Representative images of this are presented in (**b**) with the vertical lines indicating the points chosen. **c** and **d** represent the mean and SD of ln (HR) and var. ln (HR) from 100 Monte Carlo simulations, respectively

In this study the majority of events occur in the first half of the recorded time scale (Fig. 4a). The least accurate estimations of ln (HR) and var. ln (HR) occurred when weighting the extraction of points within sector 3, during which few events occurred (Fig. 4c, d). The remaining weightings differed only very marginally between weighting points in sector 1 or 2 or a uniform distribution of points.

The results of these two experiments therefore show that points extracted from the curve should be weighted towards steeper areas of the curves where there are more events and less points are required where the curves are flat and provide less information to the overall hazard ratio. As a general rule, a simple recommendation would therefore be to take points at approximately every 2% drop in survival probability in any one of the KM curves. This would ensure the event rate as a proportion of the total is relatively small between each time point although knowing this might not be possible in some cases where the number of events might be very large at any one single point in time (large vertical drops in the KM plot).

**Fig. 5:**
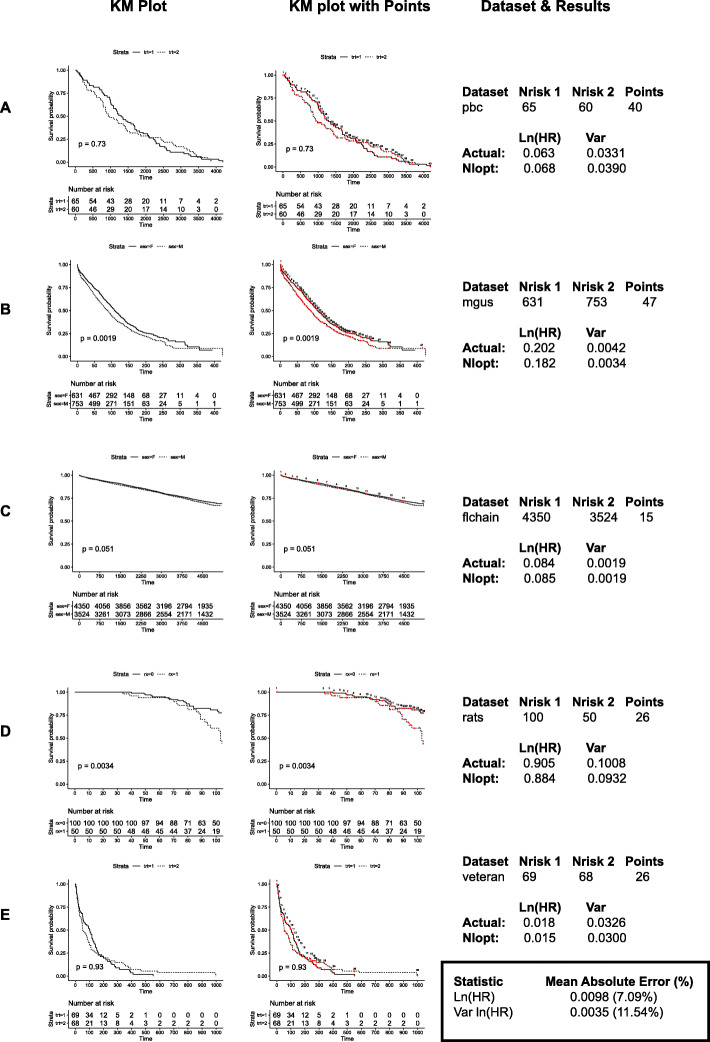
Additional validation of the nlopt method. The KM plots from five additional datasets (**a**-**e**) accessible from the R package ‘survival’ were plotted (left panel) and the ln (HR) and var. ln (HR) calculated using the rules established from Figs. 2, 3 and 4. The individual points chosen for each KM plot are overlaid on the KM plot (middle panel) and represent the exact points chosen for each dataset. The object in R used to access each dataset is included as well as the starting number at risk for each arm, the number of points used and the computed and actual values for ln (HR) and var. ln (HR) (right panel). The mean absolute error and mean % absolute error is summarised at the bottom right of the figure

To validate these rules, another set of five independent datasets were chosen to test the exact nlopt method positioning points for every 2% drop in survival. These are all IPD datasets freely accessible in the R package, ‘survival’ [27] and were chosen as they have a mix of cohort sizes (range 125–7874), study lengths (range 13–167 months) and KM survival probability end points. Figure 5 shows the KM plots in one panel and the exact points used for the analysis in the adjacent panel. The MAE for ln (HR) for this dataset was 0.0098 with a mean absolute percentage error of 7.09%. This again represents a significant improvement over the Parmar method whilst the var. ln (HR) values were similar.

We also validated the assumption of PH for each KM plot by calculating the Schoenfeld residuals (Additional File 5). In three cases, there was a non-significant relationship between these residuals and time but in the ‘rats’ dataset these were significant (*P* = 0.026) whilst the test for the ‘veteran’ dataset almost approached significance (*P* = 0.07). In both these cases, the nlopt method still produced excellent estimations of ln (HR) and var. ln (HR).

### Benchmarking of the nlopt method using an exact and non-exact *P* value

**Fig. 6:**
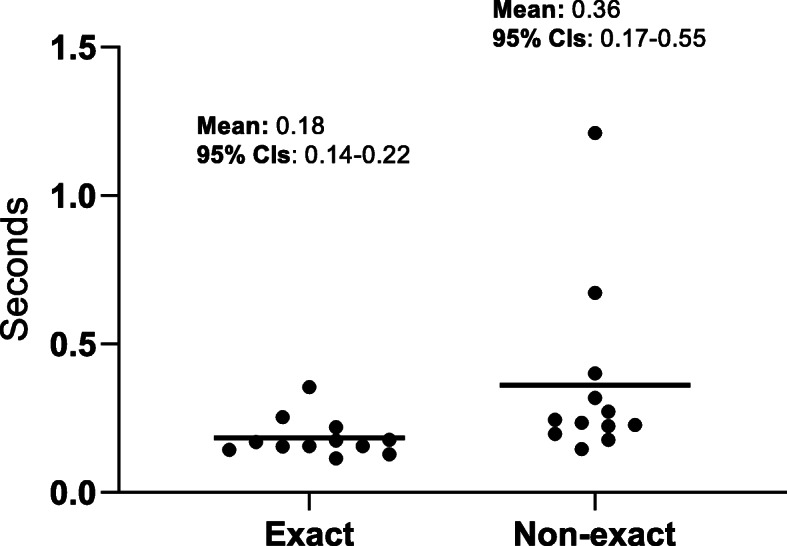
Benchmarking of the nlopt method using an exact and non-exact *P* value. The average execution time was calculated for the original 13 studies used to validate the nlopt method by taking the average from 100 iterations. The individual time for each study is plotted for the nlopt method using an exact and non-exact *P* value

The final test of the nlopt method in this study was to assess the running time required to output the results of the script. This analysis was carried out on the original series of 13 KM plots used to initially validate the nlopt method as it likely represents a more real-world situation than the simulations carried out for the point testing. The mean running time was 0.18 and 0.36 s using an exact and non-exact *P* value, respectively (Fig. 6).

## Discussion

A variety of methods have been developed to extract data from studies which fail to report summary statistics [2]. The majority of these rely on extracting time and survival probabilities from KM plots and then a set of other information that may or may not be published alongside the graph or study. In the case of Parmar et al. [8], this requires the minimum and maximum follow-up time but no other information whilst the methods by Williamson et al. [13], Hoyle et al. [14] and Guyot et al. [16] also require the number at risk. In a study comparing the accuracy in estimating ln (HR) of all four of these methods, the Guyot method was the most accurate whilst the method by Parmar least accurate. However, whereas the methods by Guyot, Williamson and Hoyle can be seen as equivalent in terms of the data input required, the Parmar method can be seen as complementary to these as it does not require the number at risk. Thus, to ensure the most accurate method is used in each scenario, Guyot should be used if the number at risk is known and the Parmar method if not.

In establishing a protocol for a meta-analysis on prognostic factors in non-small cell lung cancer, we realised that the number at risk is only infrequently included thus necessitating the use of the Parmar method. During the course of carrying out this analysis, it was realised that the *P* value is commonly stated in studies whether the number at risk is included or not, and is inherently linked to the KM survival table and thus ln (HR) and var. ln (HR). Given this, it was hypothesised that the *P* value could be used to improve the accuracy of estimating summary statistics in the absence of the number at risk.

In developing such a method, the first step is appreciating that the KM plot essentially provides all the necessary information other than the censoring pattern and time the event occurred and thus various solutions to estimate the former are required. The Parmar method assumes a constant rate of censoring across the entire study whereas the Guyot method assumes a constant rate of censoring between each published number at risk. The concept used in this method does not make assumptions about the censoring pattern but rather uses the *P* value as a fixed point and the relationship between this and the KM survival table to create a series of unknown values corresponding to the censor values at each time point to solve for. Defining the problem as such allows estimates of these unknown values to be calculated using the mathematical technique, non-linear optimisation.

Validating the nlopt method using an exact *P* value showed it is a significant improvement on the Parmar method. Reassuringly, the average ln (HR) and var. ln (HR) calculated using the Parmar method in this study (Table 3) was similar to that published in the original study suggesting there was no systemic error in the way the Parmar method was implemented. As might be expected, the Parmar method did perform well in some instances (see Addititonal file 2 and KM plots from [28, 29]), likely because the censoring was indeed constant throughout the study.

However, in the case of a non-exact *P* value, although the method generally performed well, a large error occurred in a single study when the quoted *P* value was significantly larger than the actual *P* value. Such a scenario might be expected when using non-linear optimisation with a non-exact *P* value as two types of solution can exist in optimisation methods: local and global optima. The former is an optimal solution only with respect to feasible solutions local to the objective value (i.e. the *P* value) whereas the latter can be considered to be the overall solution to the optimisation problem. In the context of the non-exact *P* value, the further the quoted *P* value is away from the actual value, the more likely only a local solution is likely to be found and thus a larger error is to be expected. Indeed, using a *P* value progressively closer to the actual value improved the estimate of ln (HR) (Additional File 4). Although the nlopt method using a non-exact *P* value in the remaining studies was more accurate than the Parmar method, since it would never be clear how far away the actual *P* value is from the quoted value in the study, we would not recommend using it in place of the Parmar method.

As has been acknowledged previously, the number and position of points is an important aspect of ensuring an optimal solution is obtained in methods extracting information from KM plots [8, 16]. The simulation experiments showed that an optimal solution varies with the number and position of points. In this particular method, the role of the time points is to ensure there are enough constraints for the non-linear optimisation to arrive at a global optimum. In some cases, even as few as 5 points (Fig. 2a) selected randomly in some of the Monte Carlo simulations proved sufficient for this when that particular set of points served as constraints which successfully minimised the objective function whilst in other cases, more points were required (Fig. 2b, c). However, since it would not be possible to determine which set of 5 points would create this scenario, a better solution would be to provide the non-linear optimisation model with enough points to ensure an optimal solution is found each time. As would be expected, the weighting experiments showed that flatter areas of the curve provide less information so points should be weighted towards steeper areas of the curve which provide more information helping to create enough unique constraints for the non-linear optimisation to arrive at this optimum. Given this, a general rule would be to take points for every 2% drop in survival probability to ensure enough points are taken for an optimal solution. In most cases this would mean between 20 and 50 points are required for any 1 KM plot. From a practical point of view, this also means an exhaustive list of points is not required, meaning the nlopt method is simple to use.

In terms of the last timepoint, this should be equal to the last point on the KM plot for both arms. Where this is not possible, i.e. one arm finishes before the other, the latter time point for either arm should be taken and the survival probablity for the other arm at its last time point used.

Although we did not directly test the relationship between the number at risk or the time length of any one individual study and estimating summary statistics, it is clear from analysing the KM plots that these are not directly contributing factors. Indeed, the largest study with over 7000 observations only required 15 points for a highly accurate estimate of the summary statistics (Fig. 5c). Moreover, longer or shorter study lengths per se do not affect the number of points required although one might expect more events (thus lower survival probabilities) in studies that have followed up their cohort for a greater length of time thus necessitating extracting more points. Our analysis has shown that the crucial determinant is ensuring the survival curve is adequately sampled along its length regardless of the size or timeframe of the study.

Importantly, the point testing simulations using a non-exact *P* value also showed that taking more points does not increase the accuracy of the estimation with an optimal solution for that particular *P* value occurring after a similar number of points. Thus, in the case of the single study where the non-exact method resulted in a large error, taking more points will likely not increase the accuracy of the estimate. Given this, we would recommend that the nlopt method using a non-exact *P* value is not used in preference to the Parmar method. However, since more studies (Table 1) publish an exact *P* value than a non-exact one, we believe the nlopt method will still be applicable in a significant number of cases.

In terms of assessing how the nlopt method would perform in studies which violate the PH assumption, this was difficult to test experimentally as only one study in the first dataset tested the PH assumption. We were able to calculate the Schoenfeld residuals for the second IPD validation datasets but since we were only able to find one dataset which violated PH and another which came close (Fig. 5d, e), we were unable to make any statistical inferences from this. Qualitatively though, in both cases, the nlopt method performed well (Fig. 5d, e). Since the nlopt method relies on a *P* value being calculated using tests that require PH [18], we would assume that it would perform better in cases where PH is not violated; however, as shown in the examples above, we believe unless the study strongly violates the assumption of PH, the nlopt method will still perform well. In such cases where the PH is clearly violated, we would expect the authors of the primary study to have stated estimates of comparing survival curves other than the hazard ratio [30].

Although the nlopt method was initially intended for studies which do not report the number at risk and thus an alternative to the Guyot method, it is of interest to see how the two compare. The MAEs presented by Guyot et al. [16] for each different scenario of data presentation are also included in Table 4. Interestingly, even in the scenario where all the information required for the Guyot method is presented, i.e. number at risk and total number of events, the nlopt method using an exact *P* value gives a more accurate estimation of ln (HR) (MAE, 0.0014 vs 0.0017) although the Guyot method is more accurate in estimating var. ln (HR) (MAE, 0.0039 vs 0.0026). However, when only one of these pieces of information is included the ln (HR) error increases (MAE, No risk: 0.036; No events: 0.028) whilst the var. ln (HR) error is variable (MAE, No risk: 0.0015; No events: 0.0065). Interestingly, Saluja et al. [15] observed in a series of oncology randomised-controlled trials, the total number of events is rarely reported even if the number at risk is, thus the Guyot method will commonly default as just the number at risk. In cases where no information is provided, the Guyot method performs worse than the Parmar method and substantially worse than the nlopt method described in this paper (Table 4).

**Table 4:** Comparison of the mean absolute error for ln (HR) and var. ln (HR) using the Parmar, Guyot and nlopt method (Exact and Non-exact *P* value) described in this study

Method	Mean Absolute Error (95% CIs)		Validation
	ln (HR)	Var ln (HR)	
1. Nlopt method: exact P value	0.014 (0.007–0.022)	0.0039 (0.0022–0.0056)	13 KM plots (this study)
2. Nlopt method: non-exact P value	0.087 (−0.036–0.210)	0.0033 (0.0010–0.0055)	
3. Parmar: this study	0.077 (0.039–0.115)	0.0038 (0.0016–0.0060)	
4. Parmar	0.079 (0.048–0.110)	0.0104 (0.0070–0.0139)	48 KM plots [8]
5. Guyot: All information	0.017 (0.002–0.122)	0.0026 (2e-5–0.1332)	6 KM plots [16]
6. Guyot: No numbers at risk	0.036 (0.003–0.242)	0.0015 (6e-6–0.0541)	
7. Guyot: No total events	0.028 (0.002–0.149)	0.0065 (2e-5–0.2497)	
8. Guyot: No additional information	0.198 (0.021–1.556)	0.1227 (6e-4–3.2501)	

Ln (HR) and var. ln (HR) reported as mean absolute error with 95% confidence intervals. The var ln (HR) for Guyot were calculated from the published standard errors by using var. = SE^2*n

In terms of deciding which method should be used to extract ln (HR) and var. ln (HR) from individual studies for use in a meta-analysis, different studies provide varying pieces of information and the most accurate method in estimating both ln (HR) and var. ln (HR) should be used in each individual case. For example, in the scenario where an exact *P* value is provided, the nlopt method described in this study could be used in all cases unless the number at risk and total number of events for each arm is known (this maximises both ln (HR) and var. ln (HR)). In the case where a non-exact *P* value is quoted, we would recommend that the nlopt method is not used at all. As such, the current, existing methods should be used depending on whether or not the number at risk is included or not.

When carrying out a meta-analysis, authors should quote the MAE associated with the method they used and could also perform a subset analysis on studies requiring extraction methods to determine if they significantly deviate from the other included studies or not.

There are several limitations of the proposed method. Firstly, if the *P* value or chi-square test statistic is not published alongside the KM plot this method cannot be used as the *P* value represents the value that the objective function has to be minimised on. As previously explained, we would also not recommend using this method when a non-exact *P* value is quoted.

As others have commented with similar methods, the quality of the initial input is important to ensure an optimal solution is obtained, particularly ensuring the points are accurately extracted from a high-quality KM plot [16].

Furthermore, although this method was primarily designed to extract ln (HR) and var. ln (HR) from Kaplan-Meier curves, which it does with good accuracy, it cannot currently output the IPD from these results. This is in contrast to the methods developed by Hoyle et al. [14] and Guyot et al. [16] whose aim was to generate the IPD from the KM plots to facilitate further secondary analysis, and not simply calculate ln (HR) and var. ln (HR) for use in a meta-analysis. This has numerous advantages, for example allowing different parametric survival models to be fitted to the IPD [3].

Indeed, future research using the nlopt method could focus on creating IPD from the current output as well as using the remaining information contained within a published study to increase the accuracy and reliability of the method. In fact, in cases where researchers want to re-capitulate IPD from a particular KM plot, any of the *P* value, hazard ratio, number at risk (where available) could be used as further constraints to improve upon existing methods as necessary.

As stated before, methods required to extract summary statistics such as ln (HR) and var. ln (HR) from published studies are only required when authors fail to report them [25], often in contrast to guidelines recommended to ensure good practice [11]. Thus, authors should be encouraged to publish as much associated information as possible when analysing survival data to facilitate these secondary analyses. Examples of such data include but are not limited to univariate hazard ratios and the corresponding variance, total number of events, number at risk values at regular intervals along the KM plot as well as exact *P* values or chi-square statistics.

## Conclusion

The objective of this article was to present a new, more accurate method for extracting ln (HR) and var. ln (HR) for aggregate meta-analyses when studies do not publish the number at risk alongside KM plots. The proposed method outperforms the current existing method and we have also produced guidance for users in choosing the position and number of points to achieve optimal estimations. In addition to the R scripts available at an online repository, a publicly-available, free-to-use web version of the software can be found at https://edgreen21.shinyapps.io/km_hr/. This is simple to use, requiring no previous programming experience, only requiring users to extract time/survival points from a KM plot using the above guidance and upload this data along with the published *P* value. The output includes the KM table and a selection of summary statistics including ln (HR) and var. ln (HR). The online version provides instructions on how to extract X,Y points from KM plots as well as a guide on how to use the web-version of the script.

## Supplementary information

**Additional file 1. **Construction of the Kaplan-Meier survival table based on the equations defined in the Methods section. The calculations for 5 timepoints are shown but this would extend for *n* number of time points. By definition, t_0_ is the start of the trial so the survival probability is 1 and there are no events or censor values. The number at risk at t_0_ is the starting number of trial participants in each arm. For clarity, the columns for survival probability, censor values, events and number at risk are only presented for Arm 1. The equations for Arm 2 would otherwise be the same. The method works by first constructing the table above assuming no censoring. The corresponding Chi-square statistic or *P* value is then used as a fixed point for the non-linear optimisation algorithm to calculate the censor values (C_1_, C_2_, .., C_n_) by iterating through possible values to satisfy the fixed value to come to an optimal solution. Ln (HR) and var. ln (HR) are then calculated based on these updated values.

**Additional file 2.** Summary of the length and status of the proportional hazards assumption in the included studies. Table of the length (months) of each study and whether the proportional hazards assumption had been checked or not from the 13 Kaplan-Meier plots used to validate the nlopt method [1, 26, 28, 29, 31–37].

**Additional file 3. **Complete table of summary statistics extracted from 13 Kaplan-Meier plots comparing methods. This includes ln (HR), variance ln (HR), the lower and upper confidence intervals (95%), the *P* value, the non-exact *P* value used with nlopt and chi-squared value. This compares the Parmar method, nlopt method from this study (both exact and non-exact *P* value) and the actual statistics extracted from each published study [1, 26, 28, 29, 31–37].

**Additional file 4. **Quoted *P* values nearer to the actual *P *values better approximate ln (HR) using the nlopt method with a non-exact *P* value. The nlopt method using a non-exact *P* value was used to calculate ln (HR) and var. ln (HR) with a range of non-exact *P* values. This started at the value quoted on the KM plot (1e-4) to values progressively closer to the actual value (4.48e-39).

**Additional file 5. **Analysis of the Schoenfeld residuals to assess the assumption of proportional hazards. The Schoenfeld residuals were calculated using the ‘cox.zph’ function in the R package ‘survival’. The assumption of proportional hazards was deemed to be violated at a *P* value ≤0.05 [38–42].

## Data Availability

The repository for the R scripts can be found at: https://gitlab.com/EdGreen21/irvinekm The web app based on the R script can be found at: https://edgreen21.shinyapps.io/km_hr/ The datasets used and/or analysed during the current study are available from the corresponding author on reasonable request.

## Abbreviations

HR: Hazard Ratio
IPD: Individual Patient Data
KM: Kaplan-Meier
MAE: Mean Absolute Error
SE: Standard Error
SD: Standard Deviation
Var: Variance
Nlopt: Non-linear optimisation
PH: Proportional Hazards
