# Supplementary material for: A non-linear optimisation method to extract summary statistics from Kaplan-Meier survival plots using the published P value
Source: BMC Med Res Methodol. 2020 Oct 30;20:269. doi: 10.1186/s12874-020-01092-x (PMC7596943; doi:10.1186/s12874-020-01092-x)
Supplement: Supplementary file 1 — Additional file 1. Construction of the Kaplan-Meier survival table based on the equations defined in the Methods section. The calculations for 5 timepoints are shown but this would extend for n number of time points. By definition, t0 is the start of the trial so the survival probability is 1 and there are no events or censor values. The number at risk at t0 is the starting number of trial participants in each arm. For clarity, the columns for survival probability, censor values, events and number at risk are only presented for Arm 1. The equations for Arm 2 would otherwise be the same. The method works by first constructing the table above assuming no censoring. The corresponding Chi-square statistic or P value is then used as a fixed point for the non-linear optimisation algorithm to calculate the censor values (C1, C2, .., Cn) by iterating through possible values to satisfy the fixed value to come to an optimal solution. Ln (HR) and var. ln (HR) are then calculated based on these updated values. [file 12874_2020_1092_MOESM1_ESM.docx]

**Additional File 1**– see end of references in main manuscript for Table title and legend.

|  | Arm 1 | | | | Total Number at Risk (NT_j_) | Total Events (ETj) | Expected Number of Events, Arm 1 (Ex1E_j_) | Expected Number of Events, Arm 2  (Ex2E_j_) |
| --- | --- | --- | --- | --- | --- | --- | --- | --- |
| Time *(t_j_)* | **Survival Probability *([S(t_j_)])*** | **Censor (C_j_)** (Unknown values) | **Events (E_j_)**  Equation (4) | **Number at Risk (N_j_)**  Equation (2) |  |  |  |  |
| t_0_ | S(t_0_) (1) | C_0_ (0) | =E_0_ (0) | =N_0_ | = N1_0_ + N2_0_ | = E1_0_ + E2_0_ | =N1_0_ * (ET_0_/NT_0_) | =N2_0_ * (ET_0_/NT_0_) |
| t_1_ | S(t_1_) | C_1_ | =N_0_-(C_0_ + E_0_)-(N_0_ -(C_0_ + E_0_)(S(t_1_)/S(t_0_) | =N_0_ - (C_0_ + E_0_) | = N1_1_ + N2_1_ | = E1_1_ + E2_1_ | =N1_1_ * (ET_1_/NT_1_) | =N2_1_ * (ET_1_/NT_1_) |
| t_2_ | S(t_2_) | C_2_ | =N_1_-(C_1_ + E_1_)-(N_1_ -(C_1_ + E_1_)(S(t_2_)/S(t_1_) | =N_1_ - (C_1_ + E_1_) | = N1_2_ + N2_2_ | = E1_2_ + E2_2_ | =N1_2_ * (ET_2_/NT_2_) | =N2_2_ * (ET_2_/NT_2_) |
| t_3_ | S(t_3_) | C_3_ | =N_2_-(C_2_ + E_2_)-(N_2_ -(C_2_ + E_2_)(S(t_3_)/S(t_2_) | =N_2_ - (C_2_ + E_2_) | = N1_3_ + N2_3_ | = E1_3_ + E2_3_ | =N1_3_ * (ET_3_/NT_3_) | =N2_3_ * (ET_3_/NT_3_) |
| t_4_ | S(t_4_) | C_4_ | =N_3_-(C_3_+ E_3_)-(N_3_ -(C_3_ + E_3_)(S(t_4_)/S(t_3_) | =N_3_ - (C_3_ + E_3_) | = N1_4_ + N2_4_ | = E1_4_ + E2_4_ | =N1_4_ * (ET_4_/NT_4_) | =N2_4_ * (ET_4_/NT_4_) |
| t_5_ | S(t_5_) | C_5_ | =N_4_-(C_4_+ E_4_)-(N_4_ -(C_4_ + E_4_)(S(t_5_)/S(t_4_) | =N_4_ - (C_4_ + E_4_) | = N1_5_ + N2_5_ | = E1_5_ + E2_5_ | =N1_5_ * (ET_5_/NT_5_) | =N2_5_ * (ET_5_/NT_5_) |
| t_n_ | S(t_n_) | C_n_ | =N_n-1_-(C_n-1_+ E_n-1_)-(N_n-1_ -(C_n-1_ + E_n-1_)(S(t_n_)/S(t_n-1_) | =N_n-1_ - (C_n-1_ + E_n-1_) | = N1_n_ + N2_n_ | = E1_n_ + E2_n_ | =N1_n_ * (ET_n_/NT_n_) | =N2_n_ * (ET_n_/NT_n_) |
|  |  | $\sum C1j$ | $\sum E1j$ |  |  |  | $\sum Ex1Ej$ | $\sum Ex2Ej$ |
|  | | | | | | **Chi-Square, Arm 1 & Arm2** | $\frac{({\sum E1j- \sum Ex1Ej)}^{2}}{\sum Ex1Ej}$ | $\frac{{(\sum E2j- \sum Ex2Ej)}^{2}}{\sum Ex2Ej}$ |
|  |  |  |  |  |  | **Chi-Square, test statistic** (Arm 1 + Arm 2) | $\frac{{(\sum E1j- \sum Ex1Ej)}^{2}}{\sum Ex1Ej}$ + $\frac{{(\sum E2j- \sum Ex2Ej)}^{2}}{\sum Ex2Ej}$ | Known value (exact or non-exact) |
|  |  |  |  |  |  | **P value** | Compare test statistic to *Χ^2^* distribution with 1 degree of freedom |  |
|  |  |  |  |  |  | **Hazard Ratio** | $\frac{{\sum E1j}/{\sum Ex1Ej}}{{\sum E2j}/{\sum Ex2Ej}}$ |  |
