# Supplementary material for: A non-linear optimisation method to extract summary statistics from Kaplan-Meier survival plots using the published P value
Source: BMC Med Res Methodol. 2020 Oct 30;20:269. doi: 10.1186/s12874-020-01092-x (PMC7596943; doi:10.1186/s12874-020-01092-x)
Supplement: Supplementary file 2 — Additional file 2. Summary of the length and status of the proportional hazards assumption in the included studies. Table of the length (months) of each study and whether the proportional hazards assumption had been checked or not from the 13 Kaplan-Meier plots used to validate the nlopt method [1, 26, 28, 29, 31–37]. [file 12874_2020_1092_MOESM2_ESM.docx]

**Additional File 2** – see end of references in main manuscript for Table title and legend.

| Article | Approximate Length of study (months) | Assumption of proportional hazards (PH) checked? |
| --- | --- | --- |
| Clark et al. (1)  *Figure 2* | 96 | Not stated |
| Bonner et al. (31)  *Figure 1* | 60 | Not stated |
| Hanley et al. (32)  *Figure 1b* | 120 | Yes, PH confirmed |
| Little et al. (28)  *Figure 2* | 1 | Not stated |
| Bonner et al. (31)  *Figure 2* | 60 | Not stated |
| Seymour et al. (33)  *Figure 3a* | 36 | Not stated |
| Rakaee et al. (34)  *Figure 4a* | 120 | Not stated |
| Rakaee et al. (34)  *Figure 4c* | 120 | Not stated |
| Bosch et al. (35)  *Figure 3a* | 25 | Not stated |
| Seligmann et al. (36)  *Figure 3a* | 22 | Not stated |
| Corrie et al. (37)  *Figure 3c* | 96 | Not stated |
| Davies et al. (29)  *Figure 2a* | 72 | Not stated |
| Breslow et al. (26) | 32 | Not stated |
|  | Median: 60 months  Range: 1-120 months |  |
