# Supplementary material for: A non-linear optimisation method to extract summary statistics from Kaplan-Meier survival plots using the published P value
Source: BMC Med Res Methodol. 2020 Oct 30;20:269. doi: 10.1186/s12874-020-01092-x (PMC7596943; doi:10.1186/s12874-020-01092-x)
Supplement: Supplementary file 3 — Additional file 3. Complete table of summary statistics extracted from 13 Kaplan-Meier plots comparing methods. This includes ln (HR), variance ln (HR), the lower and upper confidence intervals (95%), the P value, the non-exact P value used with nlopt and chi-squared value. This compares the Parmar method, nlopt method from this study (both exact and non-exact P value) and the actual statistics extracted from each published study [1, 26, 28, 29, 31–37]. [file 12874_2020_1092_MOESM3_ESM.docx]

**Additional File 3** – see end of references in main manuscript for Table title and legend.

| Article and Figure | Statistic | Actual | Parmar | Nlopt, Exact P value | Nlopt, Non-exact P value |
| --- | --- | --- | --- | --- | --- |
| Clark et al. (1)  *Figure 2* | ln(HR) | -0.54 | -0.45 | -0.58 | -0.46 |
|  | HR | 0.58 | 0.64 | 0.56 | 0.63 |
|  | Variance ln(HR) | 0.03 | 0.03 | 0.04 | 0.03 |
|  | Lower CI | 0.41 | 0.45 | 0.38 | 0.44 |
|  | Upper CI | 0.83 | 0.88 | 0.82 | 0.9 |
|  | P value (non-exact) | 0.002 (<0.01) | 0.01 | 0.002 | 0.01 |
|  | Chi-sq value | 9.1 | 7.39 | 9.1 | 6.63 |
|  |  | | | | |
| Bonner et al. (31)  *Figure 1* | ln(HR) | -0.39 | -0.34 | -0.36 | -0.34 |
|  | HR | 0.68 | 0.71 | 0.70 | 0.71 |
|  | Variance ln(HR) | 0.019 | 0.015 | 0.016 | 0.017 |
|  | Lower CI | 0.52 | 0.55 | 0.55 | 0.55 |
|  | Upper CI | 0.89 | 0.90 | 0.90 | 0.92 |
|  | P value (non-exact) | 0.005 (<0.01) | 0.01 | 0.005 | 0.009 |
|  | Chi-sq value | 7.88 | 7.62 | 7.88 | 6.63 |
|  |  |  |  |  |  |
| Hanley et al. (32)  *Figure 1b* | ln(HR) | 0.52 | 0.42 | 0.53 | 0.49 |
|  | HR | 1.69 | 1.52 | 1.70 | 1.63 |
|  | Variance ln(HR) | 0.046 | 0.048 | 0.040 | 0.044 |
|  | Lower CI | 1.11 | 0.99 | 1.15 | 1.08 |
|  | Upper CI | 2.57 | 2.34 | 2.51 | 2.46 |
|  | P value (non-exact) | 0.01 (<0.05) | 0.05 | 0.01 | 0.024 |
|  | Chi-sq value | 6.63 | 3.71 | 6.63 | 5.08 |
|  |  |  |  |  |  |
| Little et al. (28)  *Figure 2*  Article and Figure | ln(HR) | 0.058 | 0.077 | 0.068 | 0.077 |
|  | HR | 1.06 | 1.08 | 1.07 | 1.08 |
|  | Variance ln(HR) | 0.0030 | 0.0024 | 0.0029 | 0.0029 |
|  | Lower CI | 0.96 | 0.98 | 0.96 | 0.98 |
|  | Upper CI | 1.18 | 1.19 | 1.19 | 1.2 |
|  | P value (non-exact)  **Statistic** | 0.229 (0.05 < p ≤ 0.95)  **Actual** | 0.111  **Parmar** | 0.229  **Nlopt, Exact P value** | 0.125 |
|  |  |  |  |  | **Nlopt, Non-exact P value** |
| Bonner et al. (31)  *Figure 2* | ln(HR) | -0.30 | -0.22 | -0.30 | -0.26 |
|  | HR | 0.74 | 0.8 | 0.74 | 0.77 |
|  | Variance ln(HR) | 0.0191 | 0.0192 | 0.0191 | 0.0178 |
|  | Lower CI | 0.57 | 0.6 | 0.57 | 0.59 |
|  | Upper CI | 0.97 | 1.05 | 0.97 | 1 |
|  | P value (non-exact) | 0.03 (<0.05) | 0.11 | 0.03 | 0.05 |
|  | Chi-sq value | 4.71 | 2.58 | 4.71 | 3.84 |
|  |  | | | | |
| Seymour et al. (33)  *Figure 3a* | ln(HR) | 0.010 | 0.030 | 0.010 | 0.020 |
|  | HR | 1.01 | 1.03 | 1.01 | 1.02 |
|  | Variance ln(HR) | 0.0101 | 0.0099 | 0.0098 | 0.0097 |
|  | Lower CI | 0.83 | 0.85 | 0.83 | 0.84 |
|  | Upper CI | 1.23 | 1.25 | 1.23 | 1.24 |
|  | P value (non-exact) | 0.91 (0.05 < p ≤ 0.95) | 0.77 | 0.91 | 0.85 |
|  | Chi-sq value | 0.013 | 0.080 | 0.013 | 0.034 |
|  |  | | | | |
| Rakaee et al. (34)  *Figure 4a* | ln(HR) | -0.36 | -0.46 | -0.36 | -0.37 |
|  | HR | 0.7 | 0.63 | 0.7 | 0.69 |
|  | Variance ln(HR) | 0.0226 | 0.0331 | 0.0260 | 0.0249 |
|  | Lower CI | 0.51 | 0.46 | 0.5 | 0.5 |
|  | Upper CI | 0.94 | 0.9 | 0.96 | 0.94 |
|  | P value (non-exact) | 0.021 (<0.05) | 0.005 | 0.021 | 0.013 |
|  | Chi-sq value | 0.88 | 0.94 | 0.88 | 0.91 |
|  |  | | | | |
| Rakaee et al. (34)  *Figure 4c* | ln(HR) | -0.43 | -0.45 | -0.43 | -0.43 |
|  | HR | 0.65 | 0.64 | 0.65 | 0.65 |
|  | Variance ln(HR) | 0.02 | 0.02 | 0.03 | 0.03 |
|  | Lower CI | 0.48 | 0.48 | 0.48 | 0.48 |
|  | Upper CI | 0.87 | 0.87 | 0.89 | 0.89 |
|  | P value (non-exact) | 0.004 (<0.01) | 0.004 | 0.004 | 0.004 |
|  | Chi-sq value | 8.283815 | 8.283815 | 8.283815 | 8.283815 |
| Article and Figure | **Statistic** | **Actual** | **Parmar** | **Nlopt, Exact P value** | **Nlopt, Non-exact P value** |
| Bosch et al. (35)  *Figure 3a* | ln(HR) | -0.22 | -0.45 | -0.25 | -0.25 |
|  | HR | 0.8 | 0.64 | 0.78 | 0.78 |
|  | Variance ln(HR) | 0.01 | 0.02 | 0.01 | 0.01 |
|  | Lower CI | 0.60 | 0.48 | 0.64 | 0.62 |
|  | Upper CI | 1 | 0.87 | 0.95 | 0.98 |
|  | P value (non-exact) | 0.02 (<0.05) | 0.005 | 0.02 | 0.05 |
|  | Chi-sq value | 5.41 | 7.88 | 5.41 | 3.84 |
|  |  | | | | |
| Seligmann et al. (36)  *Figure 3a* | ln(HR) | -0.07 | -0.11 | -0.06 | -0.05 |
|  | HR | 0.93 | 0.9 | 0.94 | 0.95 |
|  | Variance ln(HR) | 0.039 | 0.038 | 0.034 | 0.036 |
|  | Lower CI | 0.64 | 0.62 | 0.65 | 0.66 |
|  | Upper CI | 1.37 | 1.32 | 1.35 | 1.38 |
|  | P value (non-exact) | 0.73 (0.05 < p ≤ 0.95) | 0.59 | 0.73 | 0.8 |
|  | Chi-sq value | 0.12 | 0.29 | 0.12 | 0.06 |
|  |  | | | | |
| Corrie et al. (37)  *Figure 3c* | ln(HR) | -0.223 | -0.371 | -0.248 | -0.248 |
|  | HR | 0.8 | 0.69 | 0.78 | 0.78 |
|  | Variance ln(HR) | 0.0310 | 0.0339 | 0.0375 | 0.0428 |
|  | Lower CI | 0.57 | 0.48 | 0.53 | 0.53 |
|  | Upper CI | 1.13 | 0.99 | 1.14 | 1.17 |
|  | P value (non-exact) | 0.21 (0.05 < p ≤ 0.95) | 0.06 | 0.21 | 0.239 |
|  | Chi-sq value | 1.57 | 3.54 | 1.57 | 1.39 |
|  |  | | | | |
| Davies et al. (29)  *Figure 2a*  Article and Figure | ln(HR) | -0.151 | -0.163 | -0.174 | -0.174 |
|  | HR | 0.86 | 0.85 | 0.84 | 0.84 |
|  | Variance ln(HR) | 0.0194 | 0.0238 | 0.0257 | 0.0286 |
|  | Lower CI | 0.65 | 0.62 | 0.61 | 0.61 |
|  | Upper CI | 1.13 | 1.15 | 1.15 | 1.17 |
|  | P value (non-exact) | 0.28 (0.05 < p ≤ 0.95) | 0.8 | 0.28 | 0.31 |
|  | Chi-sq value  **Statistic** | 1.167  **Actual** | 0.064  **Parmar** | 1.167  **Nlopt, Exact P value** | 1.031 |
|  |  |  |  |  | **Nlopt, Non-exact P value** |
| Breslow et al. (26) | ln(HR) | 1.191 | 1.290 | 1.168 | 0.432 |
|  | HR | 3.29 | 3.63 | 3.22 | 1.54 |
|  | Variance ln(HR) | 0.0093 | 0.0102 | 0.0182 | 0.0164 |
|  | Lower CI | 3.10 | 3.43 | 2.95 | 1.20 |
|  | Upper CI | 3.48 | 3.83 | 3.48 | 1.98 |
|  | P value (non-exact) | 4.48E-39 | 1.57E-37 | 4.47E-39 | 0.0001 |
|  | Chi-sq value | 171 | 163.9 | 171 | 15.1 |
