# Supplementary material for: A non-linear optimisation method to extract summary statistics from Kaplan-Meier survival plots using the published P value
Source: BMC Med Res Methodol. 2020 Oct 30;20:269. doi: 10.1186/s12874-020-01092-x (PMC7596943; doi:10.1186/s12874-020-01092-x)
Supplement: Supplementary file 4 — Additional file 4. Quoted P values nearer to the actual P values better approximate ln (HR) using the nlopt method with a non-exact P value. The nlopt method using a non-exact P value was used to calculate ln (HR) and var. ln (HR) with a range of non-exact P values. This started at the value quoted on the KM plot (1e-4) to values progressively closer to the actual value (4.48e-39). [file 12874_2020_1092_MOESM4_ESM.docx]

**Additional File 4** – see end of references in main manuscript for Table title and legend.

| Actual | **P value** | **Ln(HR)** | **Var ln(HR)** |
| --- | --- | --- | --- |
|  | 4.48E-39 | 1.19 | 0.0930 |
| Nlopt, non-exact | 1e-4 | 0.432 | 0.0164 |
|  | 1e-10 | 0.685 | 0.0180 |
|  | 1e-20 | 0.918 | 0.0182 |
|  | 1e-30 | 1.080 | 0.0188 |
|  | 1e-40 | 1.181 | 0.0174 |
