# Supplementary material for: A non-linear optimisation method to extract summary statistics from Kaplan-Meier survival plots using the published P value
Source: BMC Med Res Methodol. 2020 Oct 30;20:269. doi: 10.1186/s12874-020-01092-x (PMC7596943; doi:10.1186/s12874-020-01092-x)
Supplement: Supplementary file 5 — Additional file 5. Analysis of the Schoenfeld residuals to assess the assumption of proportional hazards. The Schoenfeld residuals were calculated using the ‘cox.zph’ function in the R package ‘survival’. The assumption of proportional hazards was deemed to be violated at a P value ≤0.05 [38–42]. [file 12874_2020_1092_MOESM5_ESM.docx]

**Additional File 5** – see end of references in main manuscript for Table title and legend.

| **Dataset in R Package ‘survival’** | **Associated Publication/Source** | **Correlation of Schoenfeld Residuals with Time (P value)** | **Violation of Proportional Hazards ( P≤0.05 )** |
| --- | --- | --- | --- |
| *pbc* | Therneau and Grambsch (38) | 0.179 | No |
| *mgus* | Kyle (39) | 0.118 | No |
| *flchain* | Kyle et al. (40) | 0.700 | No |
| *rats* | Mantel et al. (41) | 0.026 | Yes |
| *veteran* | Kalbfleisch and Prentice (42) | 0.070 | No |
